# Interaction of IL-6 and TNF-α contributes to endothelial dysfunction in type 2 diabetic mouse hearts

**DOI:** 10.1371/journal.pone.0187189

**Published:** 2017-11-02

**Authors:** Jonghae Lee, Sewon Lee, Hanrui Zhang, Michael A. Hill, Cuihua Zhang, Yoonjung Park

**Affiliations:** 1 Department of Health and Human Performance, University of Houston, Houston, Texas, United States of America; 2 Dalton Cardiovascular Research Center, University of Missouri-Columbia, Columbia, Missouri, United States of America; 3 Medical Pharmacology, University of Missouri-Columbia, Columbia, Missouri, United States of America; 4 Division of Sport Science and Sport Science Institute, Incheon National University, Incheon, South Korea; 5 Division of Cardiology, Department of Medicine, Columbia University Medical Center, New York, United States of America; 6 Departments of Internal Medicine, University of Missouri-Columbia, Columbia, Missouri, United States of America; 7 Physiology and Nutritional Sciences, University of Missouri-Columbia, Columbia, Missouri, United States of America; Max Delbruck Centrum fur Molekulare Medizin Berlin Buch, GERMANY

## Abstract

**Objectives:**

Inflammatory cytokines, such as tumor necrosis factor-α (TNF-α) and interleukin-6 (IL-6), are individually considered as important contributors to endothelial dysfunction in obesity and type 2 diabetes (T2D). However, their interactions in coronary arteriole endothelial dysfunction are uncertain. Therefore, this study aimed to determine the effects of TNF-α and IL-6 interactions on coronary endothelial dysfunction in experimental T2D.

**Methods:**

The studies used wild type (WT), diabetic mice (db/db), db/db null for TNF (db^TNF-^/db^TNF-^), and db/db mice treated with neutralizing antibody to IL-6 (anti-IL-6). Endothelium-dependent (acetylcholine [ACh], or luminal flow-induced shear stress) and endothelium-independent (sodium nitroprusside [SNP]) vasodilation of isolated and pressurized coronary arterioles were determined. Quantitative PCR, Western blot, and immunofluorescence staining were utilized for mechanistic studies.

**Results:**

Relative to WT, arteriolar dilation to both ACh and flow was attenuated in db/db mice and db^TNF-^/db^TNF-^. Treatment of db^TNF-^/db^TNF-^ and db/db mice with anti-IL-6 improved arteriolar dilation compared to db/db mice. Immunofluorescence staining illustrated localization of IL-6 within the endothelial cells of coronary arterioles. In db/db mice, mRNA and protein expression of IL-6 and superoxide (O_2_^-^) production were higher, but reduced by anti-IL-6 treatment. Also, in db/db mice, mRNA and protein expression of TNF-α suppressed by the anti-IL-6 treatment and the reduced expression of mRNA and protein expression of IL-6 by the genetic deletion of TNF-α both supported a reciprocal regulation between TNF-α and IL-6. Superoxide dismutase 2 (SOD2) expression and phosphorylation of eNOS (p-eNOS/eNOS) were lower in db/db mice coronary arterioles and were restored in db/db+Anti-IL-6 and db^TNF-^/db^TNF-^ mice.

**Conclusion:**

The interactions between TNF-α and IL-6 exacerbate oxidative stress and reduce phosphorylation of eNOS, thereby contributing to coronary endothelial dysfunction in T2D mice.

## Introduction

Obesity and type 2 diabetes (T2D) are frequently associated with cardiovascular diseases (CVD) [1, 2]. Vascular endothelial dysfunction, occurring at an early stage of atherosclerosis, is an important predictor of impending vascular pathology [3]. Reduced bioavailability of nitric oxide (NO), a major endothelium-dependent vasodilator, is a direct indicator of vascular endothelial dysfunction in chronic obesity and T2D [4–6]. In obesity and T2D, excess visceral fat appears to be a primary contributor to the chronic systemic low-grade inflammation linked to endothelial dysfunction and vascular inflammation [7]. Consequently, pro-inflammatory cytokines have emerged as key players in the vascular pathology associated with both obesity and T2D [7]. Although several inflammatory cytokines have been extensively studied for their contribution to endothelial dysfunction in obesity and T2D [8–13], it is less certain if some of the inflammatory cytokines may regulate the expression of others, and how interactive regulation contributes to the progression of vascular disease in T2D.

The pro-inflammatory cytokine TNF-α is a crucial regulator in insulin resistance [10, 14, 15] and endothelial dysfunction in T2D [8–10, 13, 16]. Previously, we reported that in pre-diabetic rat and diabetic mouse (db/db) models, TNF-α was responsible for impaired endothelium-dependent vasodilation in the coronary microcirculation [8–10, 17, 18]. Mechanistically, this occurred via increased production of NADPH-derived superoxide (O_2_^-^) radicals, which suppressed endothelial nitric oxide synthase (eNOS) signaling pathways and enhanced monocyte/macrophage infiltration into heart and coronary vascular tissues [8–10, 16, 19]. Pro-inflammatory IL-6, a biomarker of obesity and T2D [14, 20, 21] is a critical contributor to insulin resistance [22–24], and cardiovascular disease, including myocardial infarction and atherosclerosis [14, 25, 26]. The depletion of IL-6 signaling protected against angiotensin II-induced NO-mediated endothelial dysfunction in mouse carotid artery [11]. We previously reported that IL-6 neutralization restored impairment of endothelium-derived hyperpolarizing factors (EDHF)-mediated coronary endothelial function in a diabetic mouse model (db/db) [18]. These results provide an evidence that IL-6 plays an important role in endothelial dysfunction. Despite such a significant contribution of IL-6 and TNF-α to endothelial dysfunction as described above, a knowledge gap investigating how IL-6 and TNF-α affect each other in endothelial cell in T2D still exists. Interestingly, same previous study [18] found that IL-6 mRNA and protein expressions were significantly reduced in db^TNF-^/db^TNF-^ mice suggesting TNF-α could be a mediator of IL-6, but the interactive relationship between TNF-α and IL-6 contributing to the endothelial dysfunction of coronary microcirculation in T2D has not been clearly established yet.

Therefore, we hypothesized that IL-6 is a critical contributor to coronary endothelial dysfunction in T2D and that the interaction between IL-6 and TNF-α exacerbates endothelial dysfunction. To test the two hypotheses, we investigated: 1) Whether IL-6 contributes to the impairment of endothelial function in db/db mice; 2) Whether IL-6 and TNF-α interact to cause T2D-induced endothelial dysfunction; 3) The localization of IL-6 in vascular cells; and 4) The cellular mechanisms by which IL-6 mediates endothelial dysfunction in coronary microcirculation in db/db mice.

## Materials and methods

### Mice

Male mice at 20–24 weeks of age were used. Heterozygote controls (m Lepr^db^, WT), homozygote type 2 diabetes (Lepr^db^, db/db), and the breeding pairs of db/db mice null for TNF-α (db^TNF-^/db^TNF-^) mice were purchased from the Jackson Laboratory (Bar Harbor, Maine, USA) and maintained on a normal control chow diet. The same strain (C57BL/6J) of m Lepr^db^ and db^TNF-^/db^TNF-^ was used to match the backgrounds of Lepr^db^. The cross (db^TNF-/^db^TNF-^) of Lepr^db^ with TNF knockout was heterozygous for Lepr^db^ and homozygous for TNF knockout (TNF^-^/^-^). Genotyping of db^TNF-^/db^TNF-^ mice was performed to confirm the TNF^-/-^. db^TNF-/^db^TNF-^ mice from the second round of breeding of Lepr^db^ and TNF^-/-^ were used in subsequent experiments, and they showed the phenotypes of hyperglycemia and severe obesity, and the diabetic phenotype consistent with the penetrance of the leptin receptor mutation [16]. All procedures were approved by the University of Missouri Animal Care and Use Committee.

### Functional assessment of isolated coronary arterioles

At 20–24 weeks of age, the mice were anesthetized with pentobarbital sodium (50 mg/kg) intraperitoneal injection and sacrificed by isolation of heart. Coronary arterioles were isolated as previously described [18, 27]. In brief, individual coronary arterioles (40–100 μm diameter in situ) were dissected from mouse hearts for *in vitro* studies. Both ends of the vessels were cannulated with glass micropipettes and secured with nylon suture. Vessels were then pressurized to 60 cmH_2_O intraluminal pressure using two independent hydrostatic pressure reservoirs connected to the inflow and outflow pipettes. The cannulated vessel was bathed in a physiological salt solution (PSS) containing BSA (1%) at 37°C. Vessels not holding pressure were discarded. After development of endothelin-1-induced (0.1 nmol/L to 10 nmol/L) basal tone, the experimental interventions were performed. Endothelin-1-induced vascular tone was washed out between experiments.

Coronary arteriolar endothelium-dependent vasodilation was assessed as diameter responses to either ACh (1 nmol/L to 10 umol/L) or luminal flow (induced by pressure differences of 4–60 mmH_2_0). Endothelium-independent vasodilation was measured using the NO donor, sodium nitroprusside (SNP; 1 nmol/L to 10 umol/L). To determine the involvement of a NO-mediated mechanism in the differences in endothelium-dependent vasodilatation among WT, db/db, db^TNF-^/db^TNF-^, and db/db+anti-IL-6 mice, ACh-induced vasodilation was also performed in the presence of the NOS inhibitor NG-nitro-L-arginine-methyl ester (L-NAME; 10 umol/L, 20 min). To investigate the role of the O_2_^-^ in altered vasoactive responses, ACh-induced vasodilatory function was examined in the presence of the O_2_^-^ scavenger TEMPOL (a membrane-permeant O_2_^-^ dismutase mimetic; 1 mmol/L, 60 min incubation). To determine the role of IL-6, incubation of IL-6 (5 ng/ml) was incubated to isolated coronary arteriole in the vascular bath, and neutralization of IL-6 using anti-mouse IL-6 (0.28 mg/ml/kg, I.P. for 5 days, eBioscience) was performed.

### Immunofluorescence staining

To identify and localize IL-6 protein in coronary arterioles, transverse sections of the mouse heart were stained using markers of endothelial cells, vascular smooth muscle cells, and macrophages. Freshly isolated hearts were embedded and frozen in OCT and sectioned at 5 μm. Slides were incubated with blocking solution (10% donkey serum in PBS) and permeabilized (0.1% Triton X-100 in PBS). Primary antibodies to IL-6 (goat polyclonal 15 micro g/ml, AF-406-NA; R&D), the endothelial cell marker, von Willebrand factor (vWF; rabbit polyclonal, 1:1,000, ab6994; Abcam), smooth muscle α-actin (rabbit polyclonal, 1:800, ab5964; Abcam), or macrophage marker CD68 (rat monoclonal, 1:1000, ab53444; Abcam) were used for sequential double immunofluorescence staining. Secondary antibodies were conjugated with the fluorophores FITC or Texas red. Sections were mounted in an anti-fading agent (Slowfade gold with DAPI; Invitrogen), and then the slides were observed and analyzed with a fluorescence microscope (IX81; Olympus) with a x40 objective (0.90 numerical aperture). For negative controls, primary antibodies were replaced with goat polyclonal IgG (Abcam), rabbit polyclonal IgG (GeneTex), and rat monoclonal IgG (Abcam) isotype controls at the same concentration [28]. The specificity of the primary antibody was confirmed as the absence of immunofluorescence staining signals in the IL-6^-/-^ mice.

### Western blot analysis of protein expression

Hearts or coronary arterioles were homogenized in lysis buffer (Cellytic MT Mammalian Tissue/ Lysis/extraction Reagent; Sigma). Protein concentrations were measured using a BCA Protein Assay Kit (Pierce) and equal amounts of protein (10, 20, or 40 μg) separated by SDS-PAGE and transferred onto nitrocellulose membranes. Protein expression was detected using the appropriate primary antibody: TNF-α (1:500; R&D Systems), IL-6 (1:500; Abcam, Inc.), SOD2 (1:1,000; EMD Chemical), eNOS (1:100; Santa Cruz Biotechnology), p-eNOS (1:100; Santa Cruz Biotechnology), β-actin (1:2,000; Abcam Inc.), and corresponding secondary antibodies (1:1,000~2,000 dilution). Signals were enhanced by chemiluminescence (ECL; Amersham) and visualized with a Fuji LAS3000 densitometer. The density of protein bands obtained from the images was analyzed using Multigauge software (Fuji film). The relative densities were calculated and normalized to those of the corresponding internal reference β-actin, and then normalized to the corresponding WT control mice, which was set to a value of 1.0.

### Quantitative PCR for mRNA expression

Quantitative PCR (qPCR) was used to analyze mRNA expression for IL-6 and TNF-α in mouse cardiac tissues, using iCycler iQ5 Real-Time PCR Detection System (BioRad). Total RNA was isolated with RNeasy Fibrious Tissue Mini RNA Isolation Kit (Qiagen), and was reverse transcribed using Superscript III Reverse Transcript (Life Technologies Inc.). cDNA was amplified using qPCR Kit with SYBR^®^ Green (Life Technologies Inc.). Data were quantified by the 2^-ΔΔCT^ method as reported [29] and presented as fold change in transcripts for IL-6 or TNF-α genes (in db/db, db/db treated with neutralizing antibody to IL-6, and db^TNF-^/db^TNF-^ mice) normalized to β-actin, and compared to the WT mice (defined as 1.0 fold).

### Measurement of superoxide by electron paramagnetic resonance spectroscopy

Superoxide (O_2_)^-^ was quantified from the electron paramagnetic resonance (EPR) spectra of homogenates of mouse heart tissues. EPR peaks underwent double integration with reference to a standard curve generated from horseradish peroxidase-induced production of the anion from a standard solution of H_2_0_2_, using p-acetamidophenol as the cosubstrate [10, 30]. The values were then normalized to the mean value of WT mice.

### Data analysis

Arteriolar diameter changes to pharmacological agonists were normalized to baseline diameter (pre-drug, 60 cmH_2_O) and expressed as a percentage. Normalized diameters were averaged at each concentration of agonist used and were shown as mean ± SEM. For statistical comparison, vasomotor responses under different treatments and molecular studies between groups were performed using two-way analysis of variance (ANOVA) and one-way ANOVA, respectively. If a significant difference was obtained by ANOVA, the intergroup differences were then tested using Fisher’s protected LSD test. Significance was accepted at *p*<0.05.

## Results

### Animal characteristics

Table 1 shows the characteristics of the WT, db/db, db^TNF-^/db^TNF-^, and db/db+Anti-IL-6 mice at 20–24 weeks of age. Body weight and abdominal girth were significantly higher in db/db, db^TNF-^/db^TNF-^, and db/db+Anti-IL-6 compared to the WT mice. Non-fasting plasma glucose concentrations in the db/db, db^TNF-^/db^TNF-^, and db/db+Anti-IL-6 mice were significantly higher compared to the WT mice (Table 1). The current study utilized the new sets of animals; the animal characteristics were similar to our previous findings [18].

**Table 1:** Animal characteristics.

	WT	db/db	db/db+Anti-IL-6	db^TNF-^/db^TNF-^
**N**	11	13	6	6
**Body Weight (g)**	28.9±0.4	58.2±2.3*	56.7±2.8*	53.0±5.5*
**Abdominal Girth (cm)**	8.3±0.2	12.3±0.2*	12.6±0.2*	11.1±0.5*
**Glucose (mg/dl)**	206±10	434±18*	434±31*	464±23*

Body weight, abdominal girth, and plasma level of glucose were significantly greater in the db/db, db/db mice+Anti-IL-6, and db^TNF-^/db^TNF-^ compared to the WT mice. Differences in body weight, abdominal girth, and glucose concentration were insignificant in the db/db mice+Anti-IL-6 and db^TNF-^/db^TNF-^ compared to the db/db mice.

*p<0.05 vs. WT.

### Contribution of IL-6 to coronary endothelial function in T2D mice

**Fig 1:**
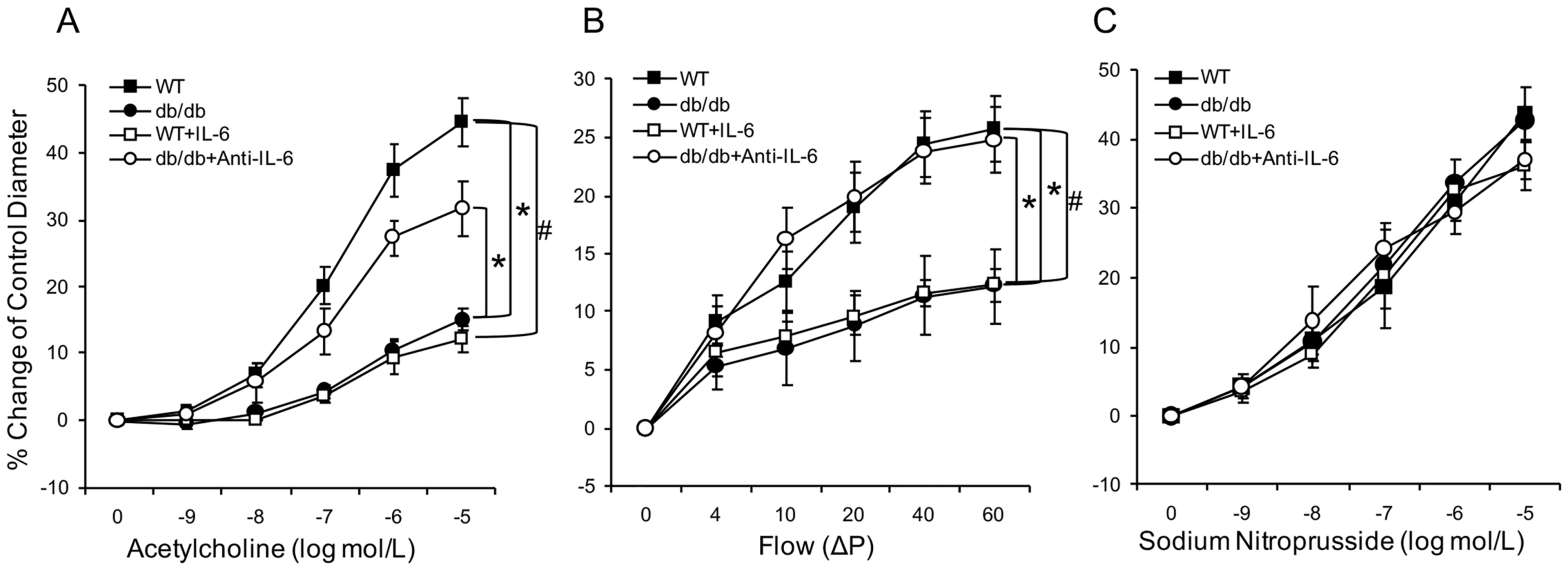
Contribution of IL-6 to endothelial dysfunction in T2D. (A) Isolated coronary arterioles from the WT (n = 11) and db/db mice (n = 13) dilated in a concentration-dependent manner to ACh. ACh-induced endothelium-dependent vasodilation was significantly reduced in the db/db mice compared to the WT mice. Incubation of IL-6 in coronary arterioles from the WT (n = 7) mice attenuated ACh-induced vasodilation. However, neutralizing antibody to IL-6 (n = 6) restored ACh-induced vasodilation of arterioles from the db/db mice. (B) Flow-induced vasodilation of coronary arterioles was impaired in the db/db mice (n = 11) compared to the WT mice (n = 10), but was enhanced by treatment with a neutralizing antibody to IL-6 (n = 6). Further, ex vivo exposure of WT coronary arterioles to IL-6 decreased flow-mediated vasodilation. (C) Vasodilation to the endothelium-independent vasodilator was not significantly different in any of the 4 groups of mice (n = 6~13). Data are shown as mean ± SEM. * *p* <.05 vs. db/db; # *p* <.05 vs. db/db+Anti-IL-6.

ACh-induced and flow-mediated endothelium-dependent vasodilation were significantly impaired in coronary arterioles isolated from the db/db mice in either a concentration or flow-dependent manner compared to the WT mice. To demonstrate the contribution of IL-6 to the impaired endothelium-dependent vasodilation in diabetic mice, coronary arterioles from WT mice were exposed to IL-6 (5 ng/ml). IL-6 attenuated endothelium-dependent vasodilation to the level of db/db, but an IL-6 neutralizing antibody to the db/db mice restored it (Fig 1A and 1B). To identify specificity for coronary endothelial cell dysfunction, smooth muscle cell-dependent vasodilatation was examined by cumulative responses to SNP. SNP-induced endothelium-independent vasodilation was identical across all mice groups (Fig 1C).

### Interactions between TNF-α and IL-6 contribute to endothelial dysfunction in T2D mice

**Fig 2:**
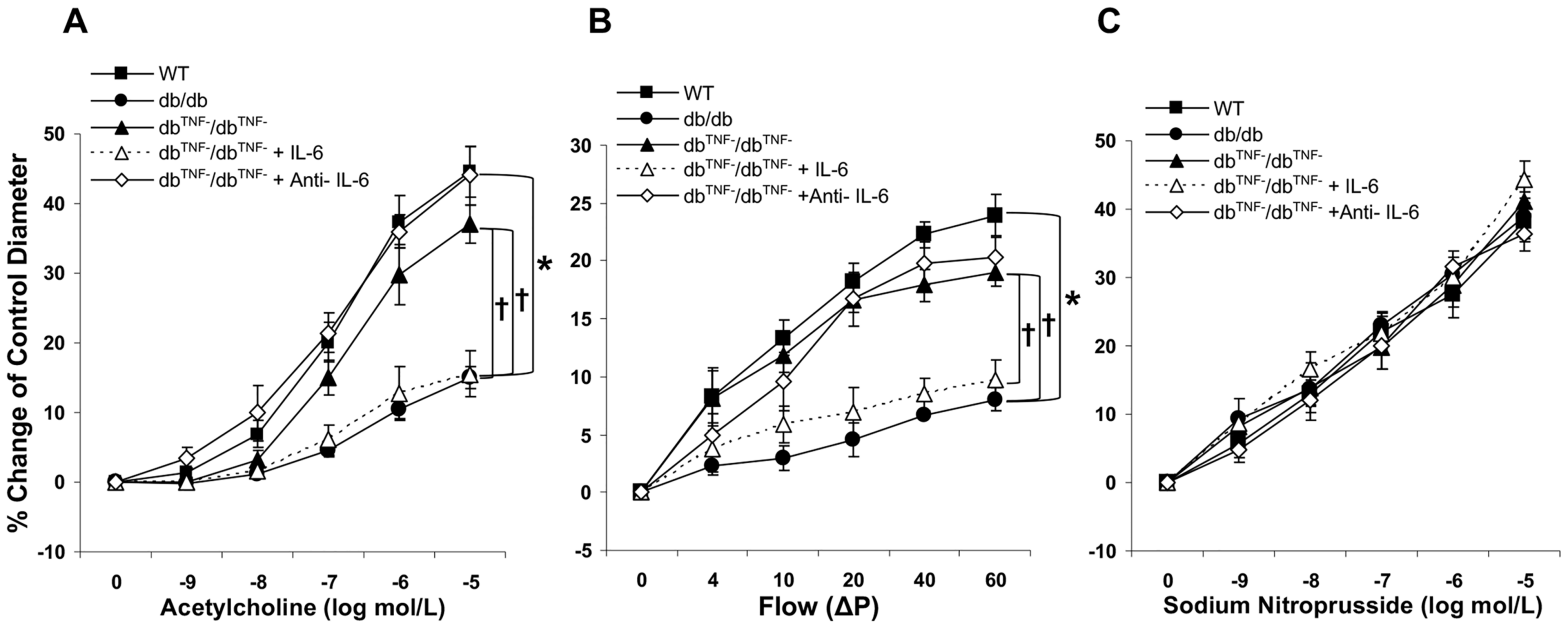
Interaction between TNF-α and IL-6 in endothelial dysfunction in T2D. (A) ACh-induced vasodilation in db/db mice null for TNF (db^TNF-^/db^TNF-^, n = 6) mice was similar to that in the WT mice and significantly higher than in the db/db mice. IL-6 attenuated ACh-induced vasodilation in db^TNF-^/db^TNF-^ mice (n = 6) to the levels observed in the db/db mice. (B) Flow-induced vasodilation in the db/db mice (n = 13) was impaired while it was preserved in db^TNF-^/db^TNF-^ mice (n = 6) which was similar to the WT mice (n = 11). Exogenous IL-6 decreased flow-mediated vasodilation of db^TNF-^/db^TNF-^ mice coronary arteriole (n = 6). (C) Endothelium-independent vasodilator responses to SNP were similar across all groups (n = 6~13). Data are shown as mean ± SEM. * *p* <.05 vs. db/db; † *p* <.05 vs. db^TNF-^/db^TNF-^.

To investigate the interactions between TNF-α and IL-6 in coronary endothelial dysfunction in T2D mice, a db/db mouse model null for TNF-α was used. Significantly improved vasodilatory responses to both ACh and intraluminal flow were observed in coronary arterioles from the db^TNF-^/db^TNF-^ mice compared to the db/db mice (Fig 2A and 2B). As these results appeared similar to those obtained in the db/db mice treated with anti-IL-6 neutralizing antibody, vessels were incubated ex vivo with IL-6. Notably, despite TNF gene knockout, IL-6 caused impaired endothelial function in db^TNF-^/db^TNF-^ vessels to levels observed in the db/db mice (Fig 2A and 2B). Treatment of the db^TNF-^/db^TNF-^ mice with anti-IL-6 neutralizing antibody restored endothelium-dependent vasodilation equivalent to those of the WT mice (Fig 2A and 2B). Endothelium-independent relaxations were similar across all groups (Fig 2C).

### Immunofluorescent localization of IL-6 to coronary arteriolar endothelial cells in T2D

**Fig 3:**
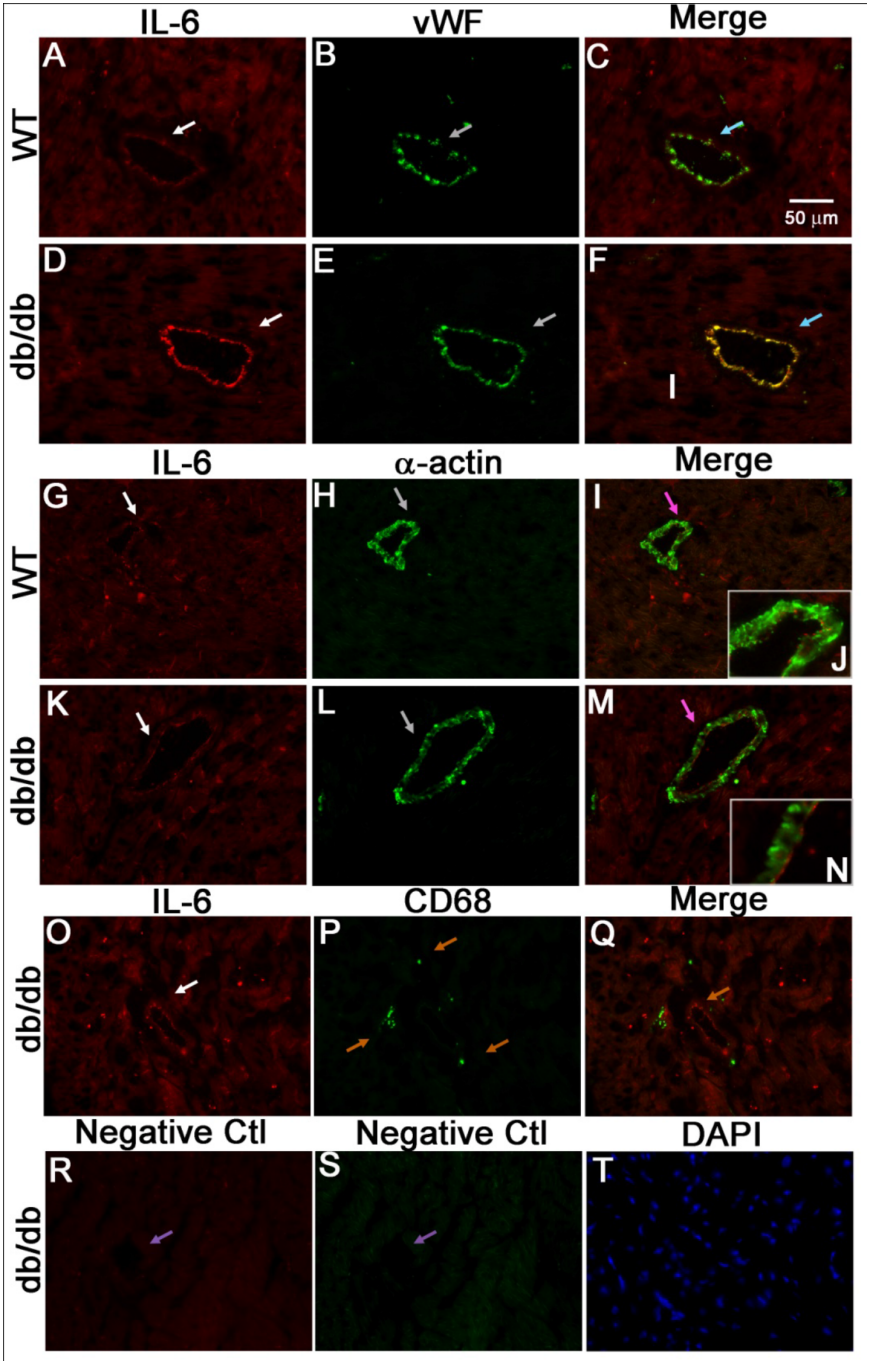
IL-6 localized to endothelial cells of coronary arterioles. Dual fluorescence combining IL-6 with markers for endothelial cells [von Willebrand factor (vWF)], vascular smooth muscle cells (α-actin) and macrophages (CD68). (A, B, and C) Dual labeling of IL-6 (red) and vWF (green) in control mouse heart tissues. (D, E, and F) Dual labeling of IL-6 (red) and vWF (green) in db/db mouse heart tissues. Arrows in C and F show the colocalization of IL-6 and endothelial cells (yellow) in the control and db/db mice. (G, H, and I) Dual labeling of IL-6 (red) and α-actin (green) in control mouse heart tissues. (K, L, and M) Dual labeling of IL-6 (red) and α-actin (green) in heart tissues of db/db. Arrows in I and M show the α-actin staining with absence of IL-6 staining in the control and db/db mice. Inserts in I (J) and in M (N) show the higher magnification indicated by arrows in I and M. O, P, and Q, dual labeling of IL-6 (red) and marker of macrophage (green) in db/db mouse heart tissues. Arrows in Q show the specific CD68 staining with absence of IL-6 staining. (R and S) Negative control: arrows show the absence of staining in vessels with control IgG and without primary antibodies. (T) Staining of nuclei with DAPI (blue) in heart tissues of the db/db mice. The immunostaining results suggest that in the hearts of type 2 diabetic mice, IL-6 is mainly localized in endothelial cells, but not in vascular smooth muscle cells and macrophages. Magnification×40. Data shown are representative of 4 separate experiments.

A dual immunostaining approach was used to identify the cellular localization of IL-6. Endothelial cells were identified by positive staining for vWF and vascular smooth muscle cells were identified by the presence of α-actin. While IL-6 was expressed in endothelial cells of coronary arterioles in the control mice (Fig 3A–3C), it was more abundant in endothelial cells of the db/db mice (Fig 3D–3F). IL-6, however, appeared to be absent in vascular smooth muscle cells of the control (Fig 3G–3J) and db/db mice (Fig 3K–3N). Further, IL-6 was absent in infiltrated macrophages in the db/db mouse heart tissues (Fig 3O–3Q). Whether IL-6 is expressed by other immune cells, including T-cell and dendritic cells [31] that have also been implicated in inflammation, remains to be determined.

### Interactive inhibitory effects between IL-6 and TNF-α in T2D

**Fig 4:**
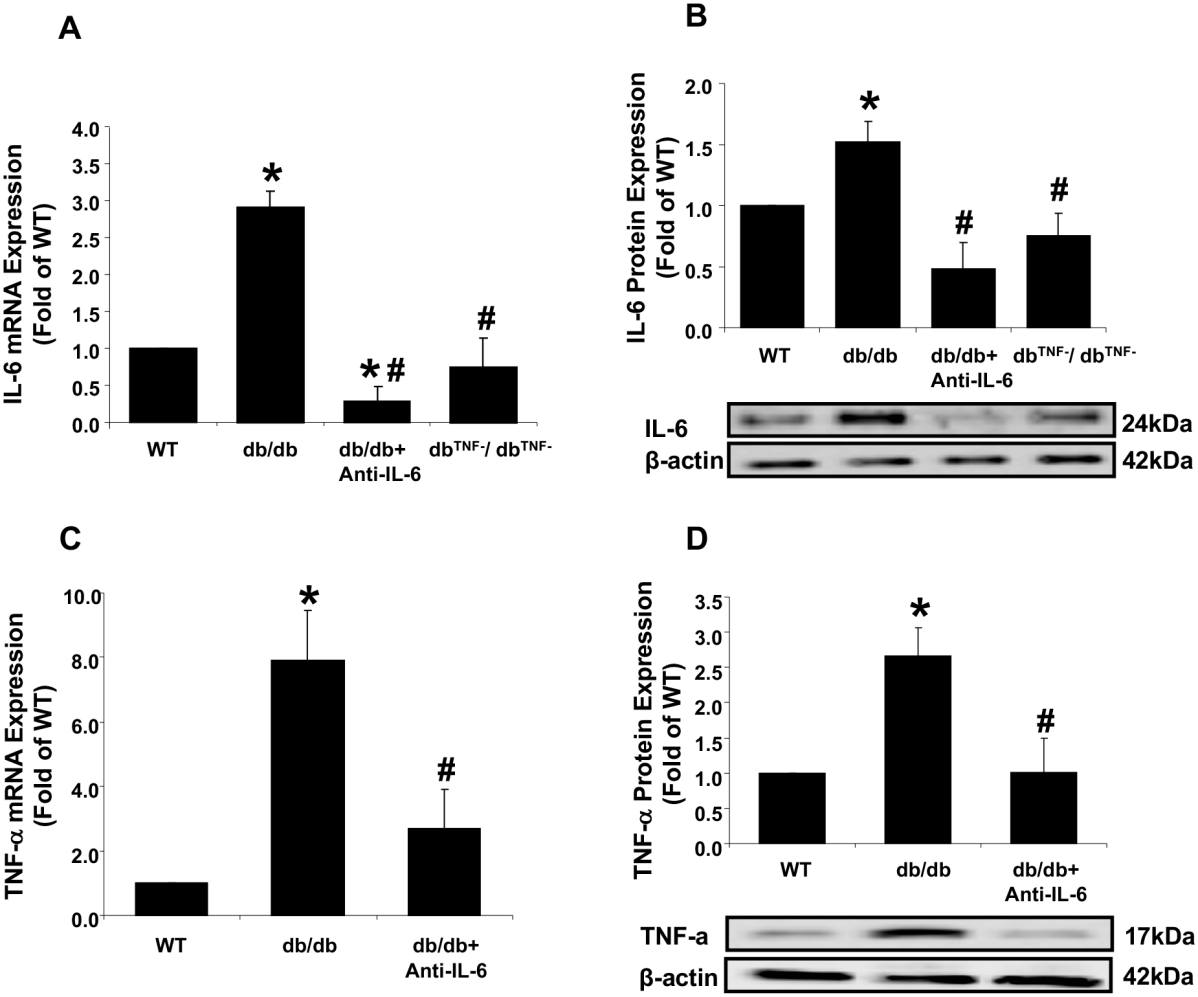
Interactive inhibition between IL-6 and TNF-α expression in diabetic endothelial cells. (A) mRNA expression of IL-6 was higher (3.6-fold) in the db/db mice compared to the WT mice, and significantly attenuated in the db/db mice treated with anti-IL-6 and in diabetic mice null for TNF-α (db^TNF-^/db^TNF-^). (B) Protein levels of IL-6 were higher in the db/db mice compared to the WT mice, but lower in the anti-IL-6 antibody-treated and db^TNF-^/db^TNF-^ mice. (C) TNF-α mRNA expression was approximately 8-fold higher in the db/db mice compared to the WT mice, but significantly lower after treatment with the IL-6 neutralizing antibody. (D) TNF-α protein levels were significantly higher in the db/db mice compared to the WT mice, but decreased in the diabetic mice receiving neutralizing antibody to IL-6 (n = 6~11). Data are shown as mean ± SEM. * *p* <.05 vs. WT; # *p* <.05 vs. db/db.

To obtain mechanistic insights, the effects of anti-IL-6 antibody treatment or TNF-α gene knockout on cytokine mRNA expression and protein levels were determined. Both IL-6 and TNF-α mRNA and protein levels were significantly greater in db/db mouse hearts compared to the WT mice (Fig 4A–4D). The reduced IL-6 mRNA and protein expression in the db^TNF-^/db^TNF-^ mice (Fig 4A and 4B) were reproduced in the current study using the new sets of animal groups from our previous study [18]. The results supported the mediation of coronary endothelial dysfunction in the db/db mice by the increased production of IL-6. In contrast, the db^TNF-^/db^TNF-^ and db/db+Anti-IL-6 treated mice demonstrated significantly attenuated mRNA and protein expression levels of IL-6 and TNF-α, respectively. Thus, treatment of anti-IL-6 and depletion of TNF-α exerted inhibitory effects on IL-6 protein and mRNA expression. Taken together, the results indicated that anti-IL-6 neutralization and TNF-α deficiency had the interactive inhibitory effects on mutual protein and mRNA expression.

### Effect of ROS on coronary endothelial dysfunction in T2D

**Fig 5:**
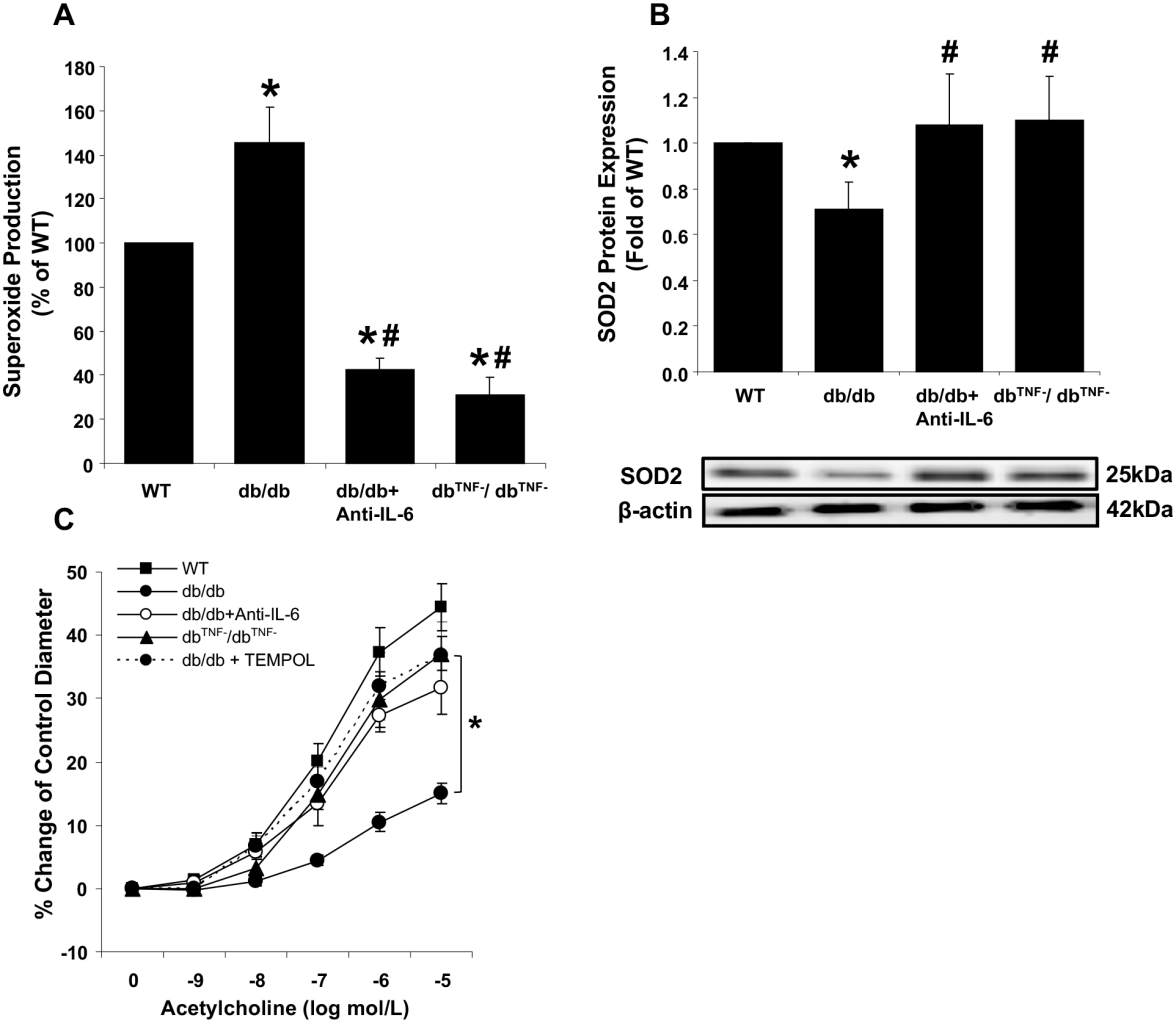
Effect of ROS on coronary endothelial dysfunction in T2D. (A) O_2_^-^ production was significantly higher in db/db mouse hearts compared to the WT mice, but attenuated in the anti-IL-6 treated db/db or null for TNF-α (db^TNF-^/db^TNF-^). (n = 6~11) (B) Protein levels of SOD2 were reduced in db/db mouse hearts, but restored to the level of the WT mice in both the anti-IL-6 db/db treated db/db mice and db/db mice null for TNF-α. (n = 6~11) (C) Incubation of TEMPOL restored endothelial function in db/db (n = 8) to the levels observed in db^TNF-^/db^TNF-^ or WT mice. Data are shown as mean ± SEM. * *p* <.05 vs. WT, # *p* <.05 vs. db/db.

It has been reported that increased ROS production directly contributes to vascular dysfunction by inhibiting eNOS signaling and decreasing NO availability [4, 5, 32]. To investigate the mechanism(s) involved in impaired vasodilation in the coronary arteriole of T2D mice, superoxide (O_2_^-^) production and a cytosolic antioxidant enzyme, SOD2, protein levels were examined. Markedly increased O_2_^-^ production was observed in diabetic mouse hearts, but was prevented by treatment with anti-IL-6 antibody or by TNF-α gene-deletion (Fig 5A). Diminished cardiac protein levels of SOD2 in the db/db mice compared to the WT mice were also observed. SOD2 protein levels were higher in the anti-IL-6 treated db/db mice or null for TNF-α compared to the db/db mice, which were similar to the levels in WT mouse hearts (Fig 5B). To provide functional evidence for diabetes-induced O_2_^-^ production causing vascular dysfunction, isolated coronary arterioles were incubated with the O_2_^-^ scavenger, TEMPOL. Eliminating O_2_^-^ significantly restored endothelium-dependent relaxation in coronary arterioles from the diabetic mice at the levels observed in the anti-IL-6 treated diabetic mice or db^TNF-^/db^TNF^ (Fig 5C). The results indicated that the elevated ROS production levels caused by the reduced SOD2 mediated coronary endothelial dysfunction in diabetes. Further, elimination of either IL-6 or TNF-α restored the impaired redox balance between the levels of ROS and the antioxidant directly responsible for endothelial dysfunction.

### Role of eNOS in endothelial dysfunction in coronary arteriole of T2D

**Fig 6:**
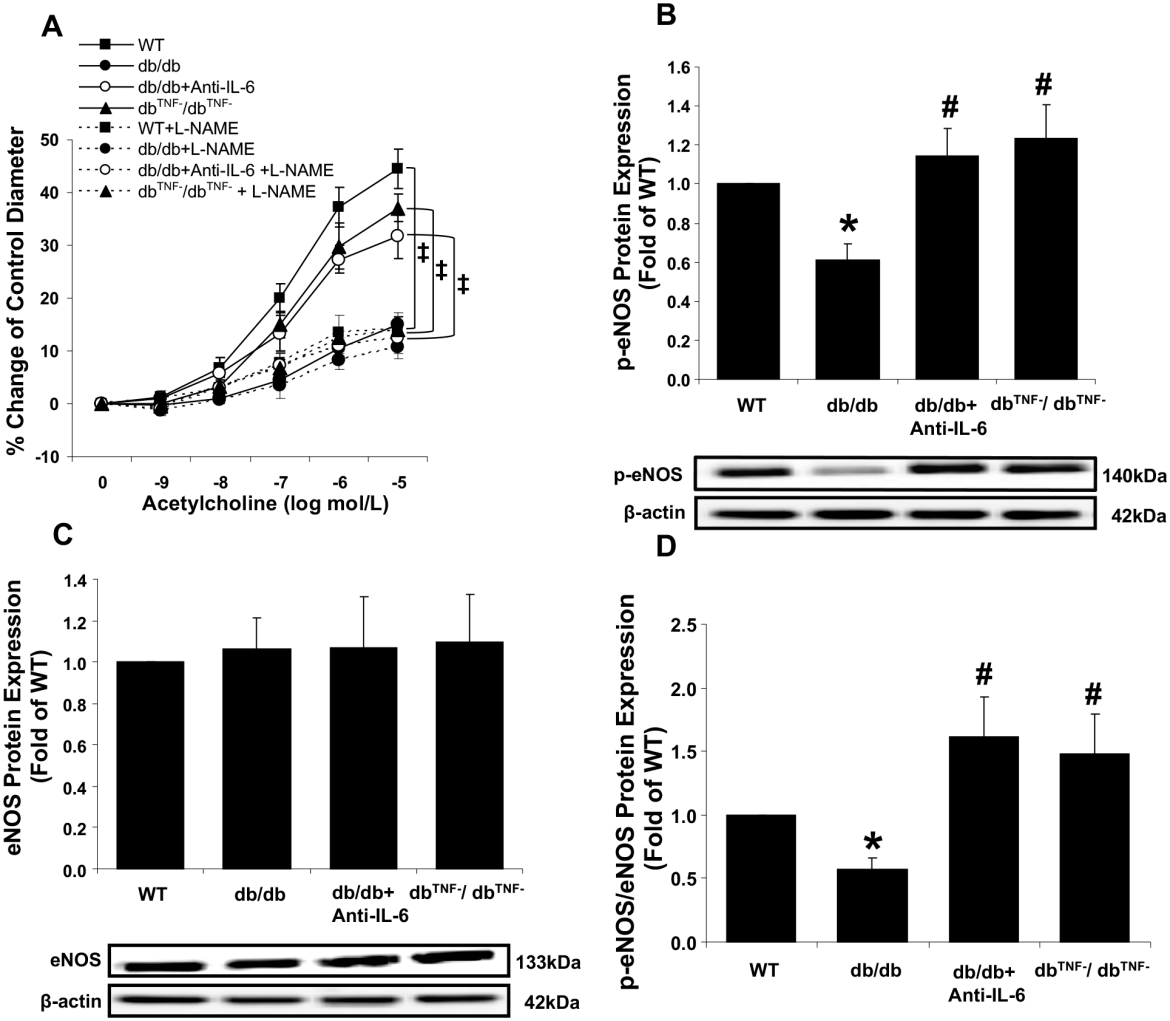
Role of eNOS in endothelial dysfunction in diabetic mouse coronary arterioles. (A) Treatment with the eNOS inhibitor, L-NAME, attenuated the improved endothelial function by inhibition of either TNF-α (n = 6) or IL-6 in the db/db mice (n = 10), but not in the db/db mice (n = 8). (B) Levels of p-eNOS protein were significantly decreased in db/db mouse hearts, but were restored in the anti-IL-6 treated db/db mice or db/db mice null for TNF-α null for at levels similar to the WT mice. (C) No differences in total eNOS protein levels were observed between groups (n = 6~11). (D) The ratio of p-eNOS to eNOS protein expression was reduced in db/db mouse hearts compared to the WT mice, while the ratios in the anti-IL-6 treated db/db mice and null for TNF-α were significantly higher than db/db (n = 6~11). Data are shown as mean ± SEM. * *p* <.05 vs. WT; # *p* <.05 vs. db/db; **‡** *p* <.05 vs. WT+L-NAME.

Figs 1 and 2 demonstrated that removal of either IL-6 or TNF-α restored endothelial dysfunction in db/db mice coronary arterioles. Since eNOS-mediated NO production has a major role in endothelium-dependent vasodilation, we also investigated whether eNOS was responsible for restored endothelial function in coronary arterioles from anti-IL-6 antibody-treated db/db and db^TNF-^/db^TNF-^ mouse hearts. We found that incubation with the eNOS inhibitor, L-NAME, significantly decreased the improved endothelium-dependent vasorelaxation in the db/db mice neutralized with anti-IL-6 and null for TNF-α (Fig 6A). The data are supported by the decreased protein levels of phosphorylated eNOS (p-eNOS) and p-eNOS/eNOS ratio in the db/db mice and the increased ratios in the anti-IL-6 antibody-treated db/db and db^TNF-^/db^TNF-^ (Fig 6B and 6D) mice. Taken together, the findings indicated that the presence of IL-6 or TNF-α in diabetic mice was associated with the reduced phosphorylation of eNOS-mediated endothelial dysfunction.

## Discussion

The results of this study provide evidence that either IL-6 inhibition or TNF deficiency has an interactive down-regulatory action on the other in type 2 diabetic coronary arterioles and it contributes to the endothelial dysfunction. The major findings of this study are as follows: 1) ACh- and flow-induced endothelium-dependent vasodilation was impaired in coronary arterioles of diabetic mice, and was mediated by IL-6 and TNF-α signaling; 2) Interactions between IL-6 and TNF-α determined the mRNA and protein expression levels for both IL-6 and TNF-α contributing to coronary endothelial dysfunction in T2D; and 3) Coronary endothelial dysfunction in T2D was regulated by IL-6 through increased production of superoxide and subsequent reduction in p-eNOS. Our findings may provide novel insight for the prevention and management of coronary artery disease associated with metabolic syndromes including obesity and type 2 diabetes.

### Role of IL-6 in coronary arteriolar endothelial dysfunction of T2D

Available evidence indicates that endothelial dysfunction is often closely related to a state of inflammation. Previously, we reported that TNF-α has a crucial role in endothelial dysfunction in T2D by activating the glycation end (AGE) product/receptor of AGE (RAGE) and the nuclear factor-kB (NF-kB) signaling pathway [9, 16, 33]. Similar to TNF-α, IL-6 has a role in the development of T2D and its complications [34, 35]. Increased serum levels of IL-6 represent an inflammatory biomarker and a risk factor of developing T2D [21, 34]. Consistent with these studies, Klover et al. reported that chronic IL-6 treatment to the mice directly impaired hepatic insulin sensitivity and that IL-6 neutralization in diet-induced obese mice improved hepatic insulin responsiveness, indicating a direct role for IL-6 in insulin resistance [22, 24]. Moreover, some studies have reported a critical role of IL-6 in vascular dysfunction; Wassmann et al. [12] observed that exogenous IL-6 treatment to wild type mice caused endothelial dysfunction in aorta through upregulation of angiotensin II type 1 receptor, while Schrader et al. [11] reported that IL-6 deficiency protected against angiotensin II-induced endothelial dysfunction in carotid arteries. Additionally, our group has provided the evidence that IL-6 is a key contributor to EDHF-mediated coronary endothelial dysfunction in T2D [18]. Despite its importance, a direct role and underlying mechanism of IL-6 in endothelial dysfunction in the coronary microcirculation of T2D have not been fully elucidated. To our knowledge the current study is the first report highlighting that IL-6 directly contributes to the coronary arteriolar endothelial dysfunction in T2D mice through enhanced oxidative stress and reduced phosphorylation of eNOS.

Our data, which demonstrate that impaired endothelium-dependent vasodilation in T2D mouse coronary arterioles was restored using IL-6 neutralization strategy (Fig 1A and 1B), provides direct evidence of the key role played by IL-6 in diabetes-induced impairment of ACh- and the physiologically relevant, flow-mediated vasodilation. We found that T2D-induced endothelial dysfunction is mediated by an impaired NO-mediated signaling pathway (Fig 6A). Previous studies, which found that exogenous IL-6 impaired endothelium-dependent vasodilator responses to ACh through blunted NO cyclic GMP signaling [36], and that IL-6 suppressed the insulin signaling pathway promoting NO production in human umbilical vein endothelial cells [23], align with our study. The results of our molecular data, which show reduced p-eNOS protein expression in the db/db mice compared to the WT mice (Fig 6B and 6D), also provide strong evidence to support the vasodilatory dysfunction in db/db mediated by a reduction of the eNOS activation responsible for NO production. This finding, in part, corresponds with previous study that incubation (10 ng/ml) of IL-6 to human aortic endothelial cells attenuated eNOS expression [37].

Finally, our immunofluorescence data showing higher expression of IL-6 protein in coronary endothelium compared to the WT mice (Fig 3A–3F) support our conclusion that IL-6-mediated endothelial dysfunction is induced by attenuated NO signaling pathways in endothelial cells. Its higher expression in the endothelial cell appears to have a direct impact on the impaired endothelium-dependent vasodilation (Fig 1A and 1B), but not on the endothelium-independent vasodilation (Figs 1C and 2C), i.e., we mainly observed IL-6 expressed in the endothelium, but not in the vascular smooth muscle cells (Fig 3G–3N).

### Interactions between TNF-α and IL-6

Pro-inflammatory cytokines, such as TNF-α and IL-6, are implicated in vascular pathophysiology. We previously determined the role of TNF-α in endothelium-dependent vascular dysfunction in T2D mouse coronary vessels [16], but the interactive relationship between TNF-α and IL-6 in vascular pathology has not been clearly identified. Turner et al. [38] reported that IL-6 mRNA expression was stimulated through TNF-α receptor I and the p38 mitogen-activated protein kinase, PI3K/Akt, and NF-kB pathway. In a previous study, we suggested that TNF-α could be an up-regulator of IL-6 at the physiological and molecular levels, i.e., restored EDHF-mediated endothelial function in the db^TNF-^/db^TNF-^ mice was attenuated in response to IL-6 incubation and expression of mRNA, and protein IL-6 was reduced in the db^TNF-^/db^TNF-^ mice [18]. Our current study reproduced these findings by utilizing new sets of animal groups (Fig 4A and 4B). In contrast, other research has proposed IL-6 as an upstream mediator of TNF-α expression in vivo and in vitro [39–42]. For example, Santo et al. (23) found that injection of LPS into IL-6-deficient mice had greatly higher plasma concentrations of TNF compared to wild type mice, while rhIL-6 treatment of macrophages inhibited TNF production. Schindler et al. [42] found that IL-6 treatment to human peripheral blood mononuclear cells suppressed TNF expression at the mRNA level.

The current study found that exposure of vessels from the db^TNF-^/db^TNF-^ mice to exogenous IL-6, reduced endothelium-dependent vasodilation in response to ACh and flow (Fig 2A and 2B). The data suggest that IL-6 has a significant role in coronary arteriolar endothelial dysfunction in the absence of TNF-α signals. This suggestion agrees with previous studies which showed that IL-6 treatment to wild type mice impaired endothelium-dependent vasodilation [12]. Notably, our molecular/cellular results (Fig 4C and 4D) showed that the inhibition of IL-6 by exogenous IL-6 neutralization suppressed mRNA and protein expressions of TNF-α in the db^TNF-^/db^TNF-^ indicating IL-6 can in fact regulate the TNF-α signaling in diabetic mice heart. Our previous study [18] reported the attenuated expression of IL-6 mRNA and protein and it was reproduced by the current study (Fig 4A and 4B), thus suggesting a possible interaction between IL-6 and TNF-α. The current follow-up study shows that the inhibition of each inflammatory cytokine, TNF-α and IL-6, has an effect on one another, in particular reciprocal down-regulatory action (Fig 4A–4D). It implicates that IL-6 and TNF-α are interactive each other to determine the endothelial dysfunction in type 2 diabetic coronary arterioles, but a further study is needed to confirm its direct relationship. It appears to be the first study to find that a reciprocal inhibitory regulation between TNF-α and IL-6 is responsible for endothelial dysfunction in T2D. Based on our novel findings of the possible interactive influence between TNF-α and IL-6, we believe that an anti-inflammatory strategy blocking IL-6 and/or TNF-α may effectively prevent T2D-mediated endothelial dysfunction in coronary microcirculation.

### Inflammation-mediated oxidative stress impairs coronary endothelial vasodilation in T2D

In concert with hyperglycemia, the inflammatory milieu producing TNF-α is considered a significant contributor to the CVD in T2D mainly through the enhanced oxidative stress. Thus, increased superoxide (O_2_^-^) production via the NAD(P)H oxidase is considered a major contributor to the mechanisms underlying impaired endothelium-dependent vasodilation in diabetes [16, 43, 44]. Our findings are consistent with the higher glucose levels (Table 1), increased TNF-α expression (Fig 4C and 4D), and enhanced superoxide production (Fig 5A) observed in the db/db mice compared to the control mice. O_2_^-^ is known to quickly react with NO, and leads to the formation of ONOO^-^ (peroxinitrite), which in turn is responsible for reducing NO biological activity and decreasing vasodilation [45, 46]. Despite many approaches, such as TNF-α in coronary arterioles and IL-6 in aortic and carotid arteries [9, 11, 12, 16], previous studies have not conclusively identified the role of IL-6 in oxidative stress-mediated coronary endothelial dysfunction in T2D.

Our data demonstrate that the diabetic condition leads to enhanced expression of IL-6 (Fig 4A and 4B) and increased generation of O_2_^-^ (Fig 5A). However, neutralization of anti-IL-6 in db/db mice showed reduced O_2_^-^ production in cardiac tissue (Fig 5A). Previous studies have shown direct effects of IL-6 on vascular oxidative stress. For example, Wassmann et al. [12] found that IL-6 was involved in upregulation of oxidative stress and impairment of endothelium-dependent vasodilation in vitro and in vivo, and Schrader et al. [11] reported that IL-6-deficient mice were resistant to angiotensin II-induced endothelial dysfunction in carotid arteries, and that vascular O_2_^-^ level and eNOS expression were preserved by angiotensin II in IL-6-deficient mice. These findings indicate the importance of IL-6 in oxidative stress-associated endothelial dysfunction in vasculature. A cytosolic antioxidant enzyme SOD2-mediated elimination of reactive oxygen species is a critical mechanism in the survival of aerobic organisms and prevention of pathological conditions [47]. Our current study also found that neutralizing IL-6 in the diabetic mice restored the expression of SOD2 protein to the levels observed in the WT mice (Fig 5B), suggesting that IL-6 is a pivotal mediator in regulating O_2_^-^ contents. Our findings show that direct exposure of TEMPOL, an O_2_^-^ scavenger, to vessels from diabetic mice reverses endothelial dysfunction, which is similar to the levels observed in the db/db+anti-IL-6 mice (Fig 5C). This result confirms that IL-6-mediated O_2_^-^ production is a primary mechanism for endothelial dysfunction in T2D coronary arterioles. The present studies, therefore, strongly suggests that IL-6 is an important contributor of T2D-mediated endothelial dysfunction, especially through elevated O_2_^-^ production and attenuated SOD2 protein expression.

## Conclusion

The interaction of TNF-α and IL-6 appears to have a pivotal role in T2D-induced endothelial dysfunction in coronary microcirculation. The effect of this interaction is mediated through enhanced oxidative stress and reduced phosphorylation of eNOS. We conclude that antagonizing TNF-α and IL-6 is an effective therapeutic approach to ameliorate T2D-associated vascular diseases.
